# eIF4A1 Inhibitor Suppresses Hyperactive mTOR-Associated Tumors by Inducing Necroptosis and G2/M Arrest

**DOI:** 10.3390/ijms23136932

**Published:** 2022-06-22

**Authors:** Luyang Han, Yuting Wu, Fangming Liu, Hongbing Zhang

**Affiliations:** State Key Laboratory of Medical Molecular Biology, Department of Physiology, Institute of Basic Medical Sciences Chinese Academy of Medical Sciences, School of Basic Medicine Peking Union Medical College, Beijing 100005, China; hlypumc@163.com (L.H.); wudadadating@outlook.com (Y.W.); lfmpumc@163.com (F.L.)

**Keywords:** *Tsc2*, *Pten*, mTOR, tumor, necroptosis, eFT226

## Abstract

Aberrantly activated mechanistic target of rapamycin (mTOR) signaling pathway stimulates translation initiation/protein synthesis and eventually causes tumors. Targeting these processes thus holds potential for treating mTOR-associated diseases. We tested the potential of eFT226, a sequence-selective inhibitor of eIF4A-mediated translation, in the treatment of mTOR hyperactive cells caused by the deletion of tuberous sclerosis complex 1/2 (TSC1/2) or phosphatase and TENsin homology (PTEN). eFT226 preferentially inhibited the proliferation of *Tsc2*- and *Pten*-deficient cells by inducing necroptosis and G2/M phase arrest. In addition, eFT226 blocked the development of *TSC2*-deficient tumors. The translation initiation inhibitor is thus a promising regimen for the treatment of hyperactive mTOR-mediated tumors.

## 1. Introduction

Hamartoma tumor syndrome can be caused by inactivating mutations of either PTEN, TSC1, or TSC2 [1,2]. As the second most frequently altered tumor suppressor in cancer, the loss of PTEN causes Cowden syndrome and endometrial carcinoma [2,3]. Loss-of-function mutations of TSC1 or TSC2 triggers the tuberous sclerosis complex, a benign tumor syndrome affecting heart, brain, lungs, kidneys, and skin [4]. Deficient TSC1 or TSC2 is also observed in hepatocellular carcinoma (HCC) and bladder cancer [5,6,7]. PTEN and TSC1/2 are major negative regulators of the AKT-mTOR pathway [8,9]. mTOR promotes translation initiation and elongation through the regulation of the eIF4E-binding proteins (4E-BPs), ribosomal protein S6 kinases (S6Ks), and eIF4F (which comprises the cap-binding protein eIF4E, the scaffolding protein eIF4G, and the RNA helicase eIF4A) [10]. These events in return control essential cellular processes including cell growth, proliferation, and survival. Inactivating mutations of TSC1/2 or PTEN causes the activation of the mTOR signaling pathway [11,12]. The mTOR inhibitor rapamycin displayed laboratory and clinical benefits for various tumors, such as TSC, bladder cancer, HCC, and endometrial carcinoma [6,12,13,14,15]. However, rapamycin exerts, primarily, a cytostatic effect. The recurrence of tumors occurred after therapy was discontinued, rendering rapamycin a lifelong therapy [16]. Besides, there are rapamycin-associated cumulative toxicities and adverse effects, including stomatitis, wound healing complications, glucose intolerance, and hyperlipidemia [17,18,19]. Given that hyperactive mTOR often leads to the dysregulation of protein synthesis, targeting cap-dependent translation is therefore an attractive cancer therapy [20,21,22].

eFT226 (Zotatifin) is the first eIF4A inhibitor to enter human clinical trials [23]. It promotes eIF4A binding to specific mRNA sequences with recognition motifs in the 5′-UTRs and interferes with the assembly of the eIF4F complex downstream of mTOR [24]. Its sensitivity correlates with the mTOR-mediated activation of eIF4A [25]. The main purpose of this study is to investigate the potential therapeutic effects and underlying mechanisms of eFT226 on *Tsc2*- and *Pten*-deficient tumors. We showed that *Tsc2*- and *Pten*-deficient mouse embryo fibroblasts (MEFs) are more sensitive to eFT226. eFT226 suppressed their proliferation by inducing necroptosis and G2/M phase arrest, and blocked growth of *Tsc2*-deficient tumors. The specific inhibition of RIPK1 signaling with necrostatin-1(Nec-1) partially reversed the suppressive effect of eFT226. These data suggest that eFT226 holds promise as a new strategy for tumors with deficient TSC1/2 or PTEN.

## 2. Materials and Methods

### 2.1. Chemicals and Reagents

eFT226 was synthesized by WuXi AppTec (Shanghai, China, purity >98%); ferrostatin-1 (Ferr-1), necrostatin-1 (Nec-1), and z-VAD-FMK (z-VAD) were obtained from SelleckChem (Houston, TX, USA); and other chemicals were obtained from Sigma-Aldrich (Burlington, MA, USA) unless specified elsewhere.

### 2.2. Cell Culture

Wild type (WT) and *Tsc2*- and *Pten*-deficient MEFs have been described previously [26,27]. Human HCC cells PLC/PRF/5 and MEFs were cultured in DMEM (Gibco, Carlsbad, CA, USA), supplemented with 10% fetal bovine serum (Gibco, Carlsbad, CA, USA) and 1% penicillin/streptomycin (Life Technologies, Carlsbad, CA, USA) in an incubator at 37 °C and with 5% CO_2_ saturated humidity.

### 2.3. Flow Cytometry

AnnexinV-647 and PI detection kit (Yeasen, Shanghai, China) was used to detect cell death. Cells were collected and washed with PBS and then resuspended in 1× binding buffer. Cells suspension (100 μL) was then transferred to a 5-mL culture tube and stained with 5 μL Annexin V-647 and 10 μL propidium iodide (PI) for 15 min at room temperature in dark. After addition of 200 μL binding buffer into each tube, the apoptotic cells were quantified using Accuri C6 flow cytometer (BD Biosciences, East Rutherford, NJ, USA). Cell Cycle Analysis Kit (Yeasen, China) was used to analyze DNA content and cell cycle profile. The cells were collected and washed with PBS and then placed in 70% ethanol overnight. Cell suspension (100 μL) was then transferred to a 5-mL culture tube and stained with 10 μL PI and 10 μL RNase for 30 min at room temperature in dark. After addition of 200 μL binding buffer into each tube, the apoptotic cells were quantified using Accuri C6 flow cytometer (BD Biosciences, East Rutherford, NJ, USA). Intracellular ROS generation was estimated using DCFH-DA (Yeasen, China). After cells were incubated with 10 μM DCFH-DA for 30 min and washed with PBS, measurements were performed using Accuri C6 flow cytometer (BD Biosciences, Franklin Lakes, NJ, USA). The ROS level is proportional to the mean fluorescence intensity (MFI) of a fluorescent probe DCFH-DA.

### 2.4. Real-Time Quantitative PCR

Total RNA was extracted from cells using Trizol (Invitrogen, Waltham, MA, USA) following the manufacturer’s instructions. RNA was reversely transcribed using PrimeScript RT Reagent Kit (Takara, Tokyo, Japan) in a total volume of 20 μL reaction, a total of 1 μg mRNA was converted into complementary DNA (cDNA). β-actin was used as internal control. Amplification was using BlastTaq^TM^ 2 × qPCR MasterMix (Applied Biological Materials, Canada. The conditions for qRT-PCR were as follows: pre-denaturation at 95 °C for 5 min, then denaturation at 95 °C for 15 s and annealing at 60 °C for 30 s. The primers used were as follows:

*β-Actin*

forward 5′-AGAGGGAAATCGTGCGTGAC-3′

reverse 5′-CAATAGTGATGACCTGGCCGT-3′;

*RIPK3*

forward 5′-GAGATGGAAGACACGGCACT-3′

reverse 5′-GGTGGTGCTACCAAGGAGTT-3′;

*RIPK1*

forward 5′-CTGTTCCCTGTGCCCAATAA-3′

reverse 5′-ATGACTCTGAAGCTGTCCTTTC-3′;

*MLKL*

forward 5′-CTGAGTTGTTGCGGGAAATCAT-3′

reverse 5′-CCGCAGACAGTCTCTCCAAGAT-3′;

*TNF-α*

forward 5′-GTCCCCAAAGGGATGAGAAGTT-3′

reverse 5′-GTTTGCTACGACGTGGGCTACA-3′;

*IL-1β*

forward 5′-CAACCAACAAGTGATATTCTCCATG-3′

reverse 5′-GATCCACACTCTCCAGCTGCA-3′

*IL-6*

forward 5′-AGATAAGCTGGAGTCACAGAAGGAG-3′

reverse 5′-CGCACTAGGTTTGCCGAGTAG-3

### 2.5. Western Blot Analysis

Cells were lysed by lysis buffer and the tumor tissue of nude mice were grinded with a homogenizer (KZ-II, Servicebio, Wuhan, China), immunoblotting was performed as previously described [28]. The primary antibodies were listed as follows. RIPK1 (rabbit, A7414, 1:1000), RIPK3 (rabbit, A5431, 1:1000), CyclinB1 (rabbit, A2056, 1:1000), CDK1 (rabbit, A0220, 1:1000), CDK4 (rabbit, A0366, 1:1000), Cdc25c (rabbit, A1672, 1:1000), c-Myc (rabbit, 9402s, 1:1000), CyclinD1 (rabbit, 55506s, 1:1000), anti-β-actin (mouse, sc-47778, 1:1000), anti-GAPDH (mouse, AC002, 1:1000), anti-vinculin (rabbit, A2752, 1:1000), anti-MLKL (mouse, 66675-1-Ig, 1:1000), anti-p-MLKL (rabbit, ab196436, 1:1000), anti-PTEN (rabbit, 9559s, 1:1000), anti-TSC2(rabbit, 4308, 1:1000), anti-mTOR(rabbit, 2983s, 1:1000), anti-p-mTOR (Ser2448) (rabbit, 2791s, 1:1000), anti-AKT (rabbit, 4691, 1:1000), anti-p-AKT (Ser473) (rabbit, 4060, 1:1000), anti-eIF4E (rabbit, 9742s, 1:1000), anti-p-eIF4E (rabbit, 9741s, 1:1000), anti-eIF4A1 (rabbit, 2490s, 1:1000), anti-eIF4G (rabbit, 2498s, 1:1000), anti-p-eIF4G (rabbit, 2442s, 1:1000), anti-eIF4B (rabbit, 3592s, 1:1000), and anti-p-eIF4B (rabbit, 3591s, 1:1000). The fluorescent secondary antibodies were diluted 1:10,000.

### 2.6. Cell Viability Assay

Cell viability was determined using CCK8 cell counting kit (Yeasen, China). Cells were seeded into 96-well plate at a density of 2 × 10^4^ cells/well and were treated with various concentrations of eFT226 for dedicated time, cells were then incubated with 10 μL of CCK8 per well for 2 h, and the absorbance at 450 nm was measured with a microplate reader (Multiskan MK3; Thermo Fisher, Waltham, MA, USA).

### 2.7. Colony Formation Assay

Cells (500 cells/well) were seeded into 6-well plates and then treated with eFT226, DMSO was used as the control. After 2 weeks, the numbers of colonies were counted under a light microscope. Images of the colonies were captured using a camera.

### 2.8. Animal Experiments

A total of 26 healthy female BALB/c nude mice aged 5–6 weeks were purchased from Beijing HFK Bioscience (Beijing, China), and all animals were housed at the Animal Center of the Institute of Basic Medical Sciences, Chinese Academy of Medical Sciences (22–24 °C, 40–60% relative humidity), food and water were freely available, and the light/dark-cycle was maintained for 12/12 h, with the light turned on at 6:00 am. For the *Tsc2*-deficient tumors, one million NTC/T2 deficient (*Tsc2^−/−^, Tp53^−/−^*) cells [29] and two million PLC/PRF/5 cells were inoculated into the right flanks of the mice, respectively. When the tumor size reached 80–100 mm^3^, the mice were randomly divided into four groups using random number table: control group (5% dextrose in water) (n = 8 for *Tsc2^−/−^*, *Tp53^−/−^*MEFs, n = 5 for PLC/PRF/5 cells) and eFT226 group (1 mg/kg) (n = 8 for *Tsc2^−/−^*, *Tp53^−/−^*MEFs, n = 5 for PLC/PRF/5 cells), respectively, once weekly for three weeks. The mice were weighed every other day. Then the mice were sacrificed, and the tumors were collected.

### 2.9. Histology Study

Tumor samples harvested from tumor-bearing nude mice were fixed in formalin, sectioned, and stained with hematoxylin and eosin (H&E) following the standard protocols. Immunohistochemistry for detection of Ki67 was performed according to standard protocols.

### 2.10. Statistical Analysis

GraphPad Prism V.8 was used for statistical analysis (San Diego, CA, USA). Data are presented as the means ± SD. The unpaired Student’s *t*-test was used for the comparison between two groups. One-way ANOVA was used for comparing multiple groups of quantitative data. (* *p* < 0.05, ** *p* < 0.01, *** *p* < 0.001). All data are representative of at least three independent experiments.

## 3. Results

### 3.1. Tsc2- and Pten-Deficient Cells Are More Sensitive to eFT226 Treatment

**Figure 1:**
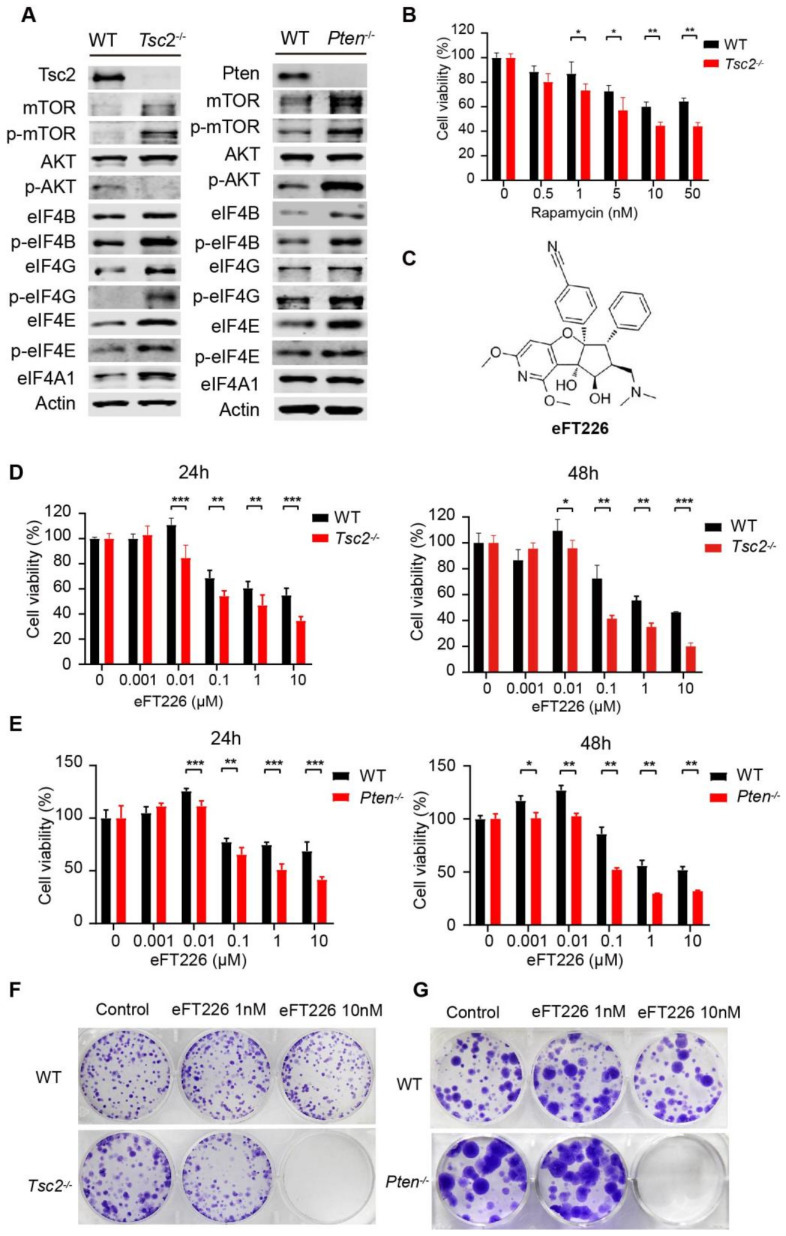
*Tsc2*- and *Pten*-deficient cells are more sensitive to eFT226 treatment. (**A**) Immunoblot analysis of mTOR and its downstream translation initiation factors in *Tsc2*- and *Pten*-deficient MEFs. (**B**) *Tsc2*-deficient MEFs were treated with rapamycin for 48 h. (**C**) Chemical structure of eFT226. *Tsc2*- (**D**) and *Pten*-deficient (**E**) MEFs were treated with eFT226 for 24 h and 48 h at the indicated concentration in quintuplicate. Cell viability was assessed by CCK-8 assay. Colony formation of *Tsc2*- (**F**) and *Pten*-deficient (**G**) MEFs treated with eFT226 or its solvent DMSO in triplicate. (**B**,**D**,**E**) are the results of triplicate experiments. (**F**,**G**) are one set images from duplicate experiments. Error bars represent mean ± SD. * *p* < 0.05; ** *p* < 0.01; *** *p* < 0.001.

Since the hyperactivation of mTOR and translation initiation factors were presented in *Tsc2*- and *Pten*-deficient MEFs (Figure 1A), we examined the effect of eFT226 on the viability of mTOR-activated cells. *Tsc2*-deficient MEFs were more sensitive to rapamycin as previously reported [30,31] (Figure 1B). Biochemical analysis shows that eFT226 (Figure 1C) [23], a selective eIF4A1 inhibitor, preferentially attenuated the proliferation and colony formation assay of *Tsc2*- and *Pten*-deficient MEFs (Figure 1D–G). These findings suggested that *Tsc2*- and *Pten*-deficient cells are more sensitive to eFT226 treatment.

### 3.2. eFT226 Inhibits Translation Initiation Factors

**Figure 2:**
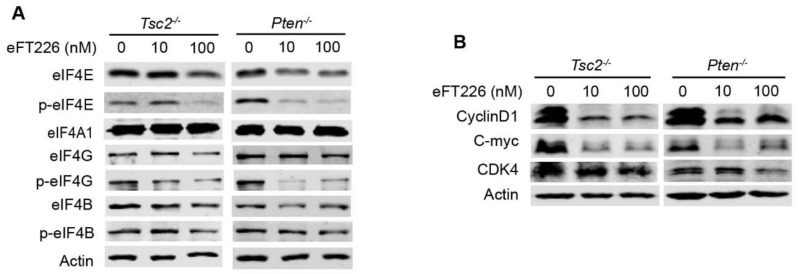
eFT226 inhibits translation initiation factors. Immunoblotting of translation initiation factors (**A**) and their targeted genes (**B**) in *Tsc2*- and *Pten*-deficient MEFs treated with eFT226 for 48 h.

eFT226 inhibited the activation of mTOR downstream translation initiation factors, except for the expression of eIF4A in *Tsc2*- and *Pten*-deficient MEFs (Figure 2A). Oncogenic and survival factors including MYC, cyclin-dependent kinases 4 (CDK4), and cyclin D1 have highly structured 5′-UTR regions that are thus more dependent on eIF4A RNA helicase enzyme activity to drive mRNA translation [32,33]. eFT226 reduced MYC, CDK4, and cyclin D1 in *Tsc2*- and *Pten*-deficient MEFs (Figure 2B).

### 3.3. eFT226 Triggers Necroptosis in Tsc2- and Pten-Deficient Cells

**Figure 3:**
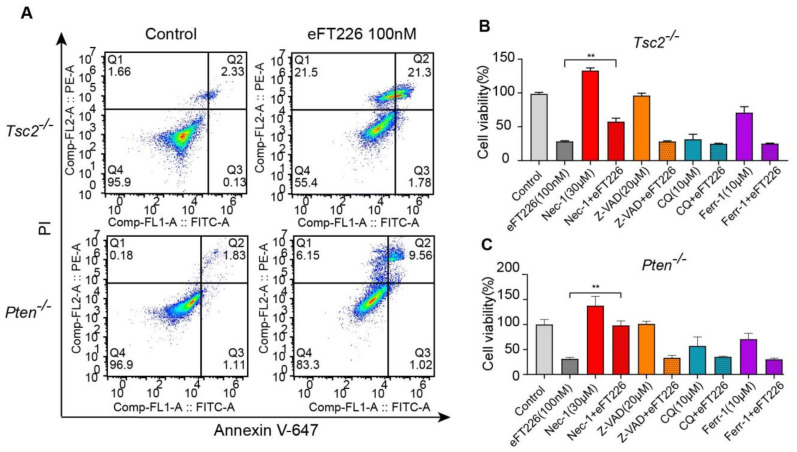
eFT226 triggers necroptosis of *Tsc2*- and *Pten*-deficient cells. (**A**) *Tsc2*- and *Pten*-deficient MEFs were treated with eFT226 (100 nM) or its solvent DMSO in triplicate for 48 h. Cells were stained with Annexin V-647 and propidium iodide (PI) for dead cells then analyzed with Accuri C6 flow cytometer. *Tsc2*- (**B**) and *Pten*-deficient MEFs (**C**) were treated with or without eFT226 (100 nM) and/or various inhibiters in quintuplicate for 48h. Cell viability was checked by CCK-8 assay. Values are mean ± S.D. of 3 independent experiments in comparison with the control. ** *p* < 0.01.

**Figure 4:**
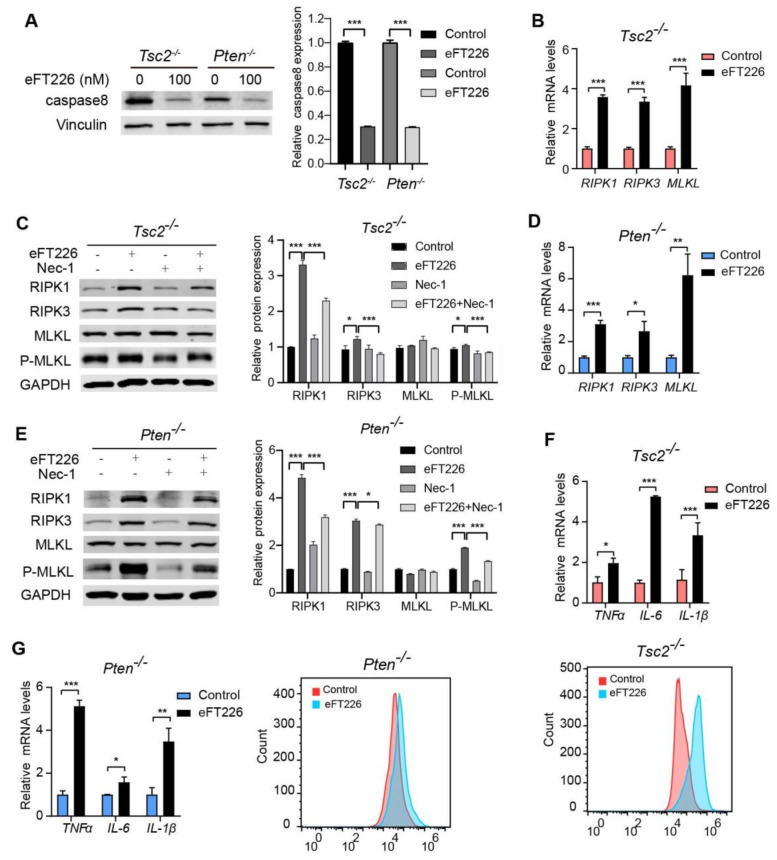
eFT226 activates RIPK1/RIPK3/MLKL pathway. (**A**) Immunoblot analysis of caspase8 protein in *Tsc2*- and *Pten*-deficient MEFs after treatment with eFT226 (100 nM) for 48 h (**left**). Quantitative analysis of protein band density was shown in right panel. (**B**) Effects of *RIPK1, RIPK3,* and *MLKL* mRNA expression in the *Tsc2*- deficient MEFs was determined by RT-PCR. (**C**) The levels of necroptosis proteins were analyzed by immunoblotting after eFT226 (100 nM) treatment with or without Nec-1 in *Tsc2*- deficient MEFs (**left**). Quantitative analysis of protein band density was shown in right panel. (**D**) mRNA expression level of *RIPK1, RIPK3,* and *MLKL* in the *Pten*-deficient MEFs was determined by RT-PCR. (**E**) The levels of necroptosis proteins were analyzed after eFT226 (100nM) treatment with or without Nec-1 in *Pten*-deficient MEFs (**left**). Quantitative analysis of protein band density was shown in right panel (**right**). *Tsc2*- (**F**, **top**) and *Pten*-deficient MEFs (**G**, **left**) were treated with eFT226 (100 nM) for 48 h and mRNA levels of *TNF-α*, *IL-6*, and *IL-1β* were measured by RT-PCR. ROS levels were measured in *Tsc2*- (**F**, **bottom**) and *Pten*-deficient MEFs (**G**, **right**) by flow cytometry using DCFH-DA probe after treatment with eFT226 (100 nM) in triplicate for 48 h. All quantitative RT-PCR reactions were performed at least in triplicate. Values are mean ± S.D. of 3 independent experiments in comparison with the control. * *p* < 0.05; ** *p* < 0.01; and *** *p* < 0.001.

Decreased cell viability may be due to decreased cell proliferation or increased cell death. Increased cell death was detected by Annexin V-647/PI staining in eFT226-treated *Tsc2*- and *Pten*-deficient MEFs (Figure 3A). To determine the mode of eFT226-induced cell death, we treated cells with inhibitors for autophagy (chloroquine, CQ), apoptosis (Z-VAD-FMK), ferroptosis (ferrostatin-1, ferr-1), and necroptosis (necrostatin-1, Nec-1). Notably, only Nec-1 alleviated the cell death caused by eFT226 in *Tsc2*- (Figure 3B) and *Pten*-deficient MEFs (Figure 3C). Caspase8 inhibition is known to switch apoptosis to necroptosis. eFT226 indeed decreased caspase8 expression in *Tsc2*- and *Pten*-deficient MEFs (Figure 4A) and enhanced the mRNA (Figure 4B) and protein (Figure 4C) levels of critical mediators of the necroptotic signaling pathway, including RIPK1, RIPK3, and MLKL in *Tsc2*-deficient MEFs. The same results were obtained in *Pten*-deficient MEFs (Figure 4D,E). Necroptosis is an inflammatory response that is accompanied with accumulated ROS and inflammatory cytokines [34,35]. The expressions of *TNF-α*, *IL-6*, and *IL-1β* mRNA were increased (Figure 4F, **top**) and the ROS level was higher (Figure 4F, **bottom**) in *Tsc2*-deficient MEFs with eFT226 treatment. The same results were obtained in *Pten*-deficient MEFs (Figure 4G).

### 3.4. Nec-1 Partially Reversed Necroptosis Caused by eFT226

**Figure 5:**
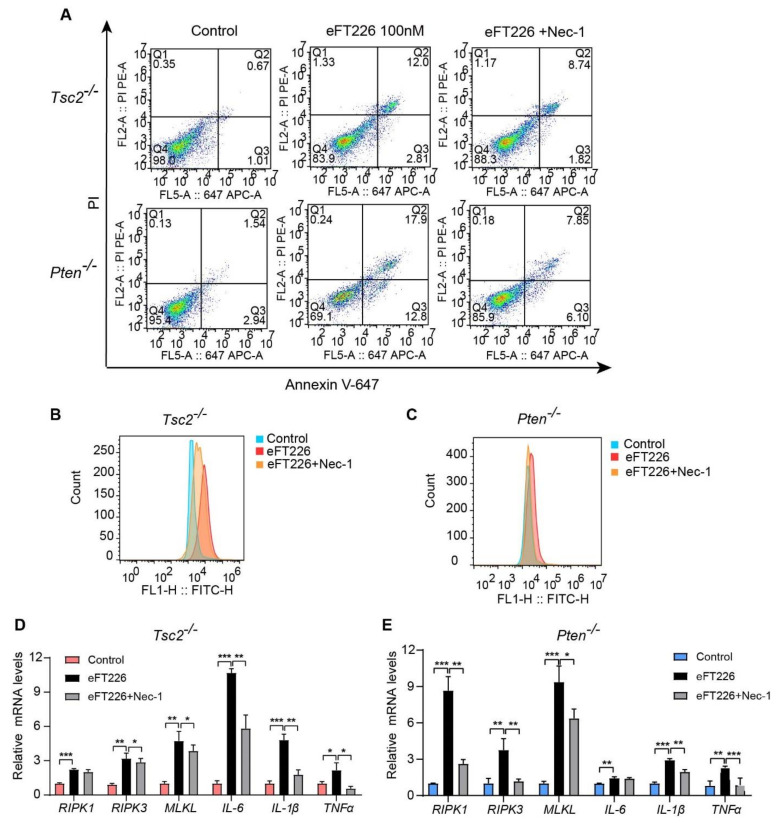
Nec-1 partially reverses necroptosis caused by eFT226. (**A**) After treatment with eFT226 (100 nM) with or without Nec-1 (30 μM), *Tsc2*- and *Pten*-deficient MEFs were stained with Alexa Fluor-647 and PI and analyzed by Accuri C6 flow cytometer. ROS levels were measured in *Tsc2*- (**B**) and *Pten*-deficient MEFs (**C**) by flow cytometry using the DCFH-DA probe after treatment with eFT226 (100 nM) in triplicate for 48 h. Necroptosis and proinflammatory cytokines mRNA were determined by RT-PCR in *Tsc2*- (**D**) and *Pten*-deficient MEFs (**E**). Experiments were repeated 3 times with triplicate wells. Error bars represent mean ± SD. * *p* < 0.05; ** *p* < 0.01; and *** *p* < 0.001.

Next, we asked whether the inhibition of necroptosis prevented cell death induced by eFT226. *Tsc2*- and *Pten*-deficient MEFs were pretreated with Nec-1 and then co-incubated with eFT226. Nec-1 partially reversed eFT226-induced cell death (Figure 5A), ROS accumulation (Figure 5B,C), and inflammatory cytokines expression (Figure 5D,E). Moreover, Nec-1 partly reversed the enhanced expression of RIPK1, RIPK3, and p-MLKL caused by eFT226 at mRNA (Figure 5D,E) and protein levels (Figure 4C,E). Taken together, eFT226 induces necroptosis in *Tsc2*- and *Pten*-deficient MEFs.

### 3.5. eFT226 Induces G2/M Arrest in Tsc2- and Pten-Deficient Cells

**Figure 6:**
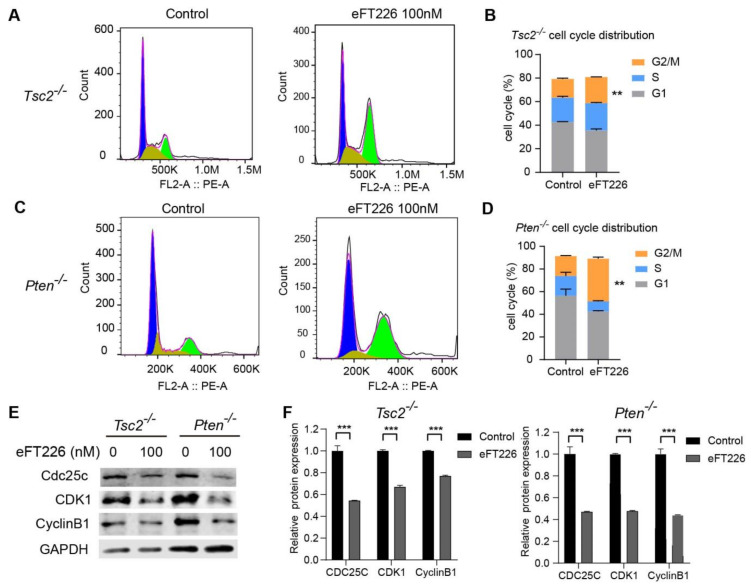
eFT226 induces G2/M arrest in *Tsc2*- and *Pten*-deficient cells. *Tsc2*- (**A**,**B**) and *Pten*-deficient MEFs (**C**,**D**) were treated with eFT226 (0 nM, 100 nM) for 48 h in triplicate and cell cycle distribution was measured by PI staining followed by flow cytometry. The populations in G1, S and G2/M stages are shown in blue, yellow and green, respectively. (**E**) *Tsc2*- and *Pten*-deficient MEFs treatment with eFT226 (100 nM) for 48 h were immunoblotted with anti-Cdc25c, anti-CDK1, anti-cyclinB1 antibodies. (**F**) Densitometric quantification of representative immunoblots from E. Values are mean ± S.D. of 3 independent experiments in comparison with the controls. ** *p* < 0.01; *** *p* < 0.001.

**Figure 7:**
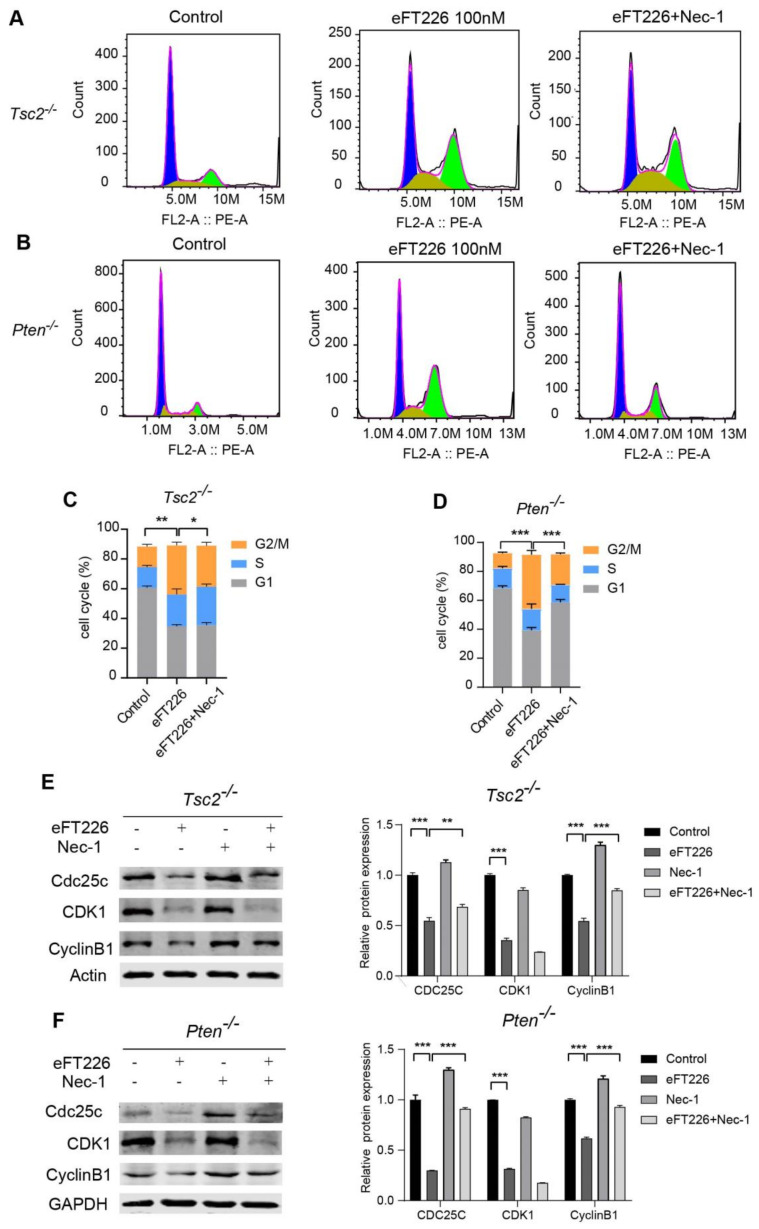
Nec-1 partially reverses G2/M arrest caused by eFT226 in *Tsc2*- and *Pten*-deficient cells. (**A**–**D**) *Tsc2*- (**A**,**C**) and *Pten*-deficient MEFs (**B**,**D**) were treated with eFT226 (100 nM) with or without Nec-1 (30 μM) for 48 h in triplicate and cell cycle distribution was measured by PI staining followed by flow cytometry. The populations in G1, S and G2/M stages are shown in blue, yellow and green, respectively. (**E**,**F**) *Tsc2*- and *Pten*-deficient MEFs were treated with eFT226 (100 nM) with or without Nec-1 for 48h and then subjected to immunoblotting. Quantitative analysis of the band density was shown in right panel. Error bars represent mean ± SD. * *p* < 0.05; ** *p* < 0.01; and *** *p* < 0.001.

eFT226 increased the percentage of *Tsc2*- (Figure 6A,B) and *Pten*-deficient MEFs (Figure 6C,D) in the G2/M phase. The markers of the G2/M checkpoint, including Cdc25c, Cyclin B1, and CDK1, were decreased after eFT226 treatment (Figure 6E,F). These results suggest that eFT226 caused G2/M arrest in *Tsc2*- and *Pten*-deficient MEFs. Next, we explored the role of necroptosis in eFT226-triggered G2/M arrest. eFT226-induced G2/M arrest was restored by the necroptosis inhibitor Nec-1 in *Tsc2*- and *Pten*-deficient MEFs (Figure 7A–D). Furthermore, eFT226-suppressed the expression of Cdc25c and CyclinB1 but not CDK1, which was recovered after Nec-1 treatment (Figure 7E,F). These data indicate that necroptosis plays vital roles in G2/M arrest caused by eFT226.

### 3.6. eFT226 Suppresses Tsc2-Deficient Tumor Growth

**Figure 8:**
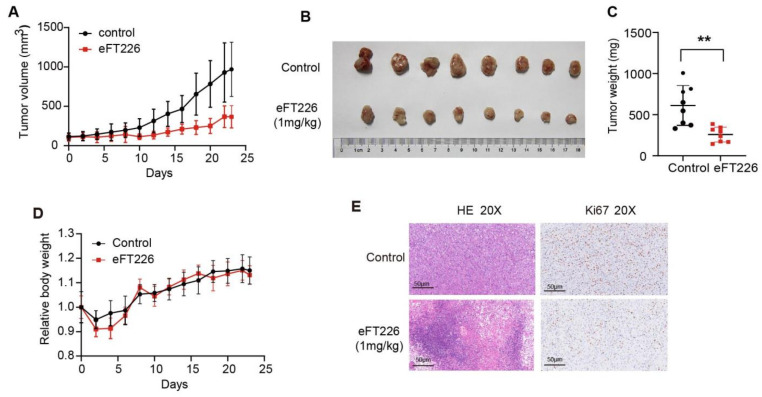
eFT226 suppresses *Tsc2*-deficient tumor growth. Nude mice were subcutaneously inoculated with *Tsc2*-deficient MEFs and then treated with vehicle (5% dextrose in water) (n = 8) or eFT226 (1 mg/kg, 1 times/week) (n = 8). (**A**) Tumor volume of nude mice was recorded every 2 days. (**B**) A representative picture of a tumor is shown as an inset. (**C**,**D**) The measurement of tumor weight and body weight. (**E**) Representative images of H&E and immunohistochemistry for Ki-67 from tumors. (Scale bars: 50 μm). Error bars represent mean ± SD. ** *p* < 0.01.

**Figure 9:**
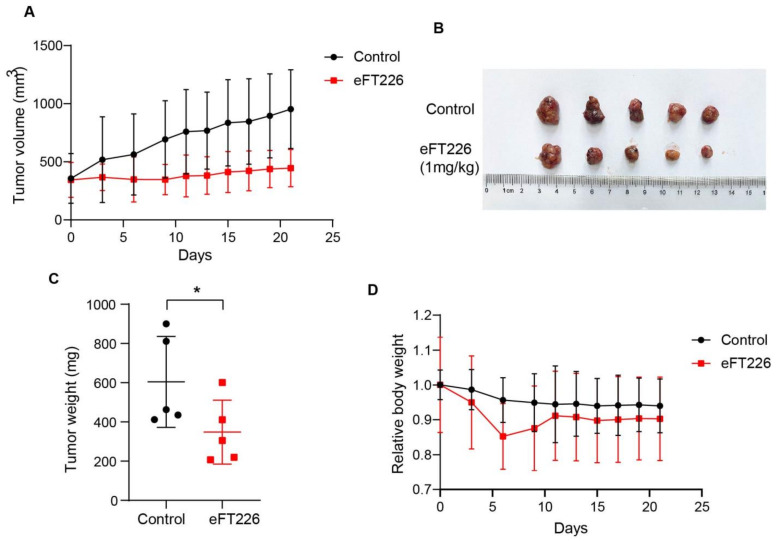
eFT226 suppresses PLC/PRF/5 tumor growth. Nude mice bearing subcutaneous tumors derived from PLC/PRF/5 cells were i.p. administered with vehicle control (5% dextrose in water) (n = 5) or eFT226 (1 mg/kg, 1 times/week) (n = 5) for 3 weeks. Tumor volumes (**A**), photographs of tumors (**B**), tumor weights (**C**), and mouse body weights (**D**) are shown. Error bars represent mean ± SD. * *p* < 0.05.

The tumor-bearing nude mouse model is a widely used pre-clinical model for the screening of drugs in vivo [29]. The NTC/T2 deficient cell line was reported to induce *Tsc2^−/−^* tumors in nude mice [29]. To check the efficacy of eFT226 in hyperactive mTOR-associated tumors, we first treated nude mice bearing *Tsc2*-deficient tumors with 1 mg/kg eFT226 once per week. eFT226 blocked tumor growth, as indicated by a reduced tumor volume and weight in the treatment group (Figure 8A–C), without an effect on the body weight of mice (Figure 8D). Besides, H&E staining showed that the eFT226 group displayed necrotic areas infiltrated with inflammatory cells, and immunohistochemistry staining displayed that the Ki-67 expression level, a cell-proliferation marker, was down-regulated in the eFT226 group (Figure 8E). In addition, a PLC/PRF/5 xenograft mouse model was also established. As shown in Figure 5, eFT226 dosing for three weeks suppressed tumor volume and weight in PLC/PRF/5 cell-bearing mice (Figure 9A–C). There was a slight decrease in the body weight of the eFT226-treated group compared to the vehicle-treated group (Figure 9D). Overall, these results indicate that eFT226 hinders *Tsc2*-deficient tumor growth.

## 4. Discussion

Activating mutations of proto-oncogenes such as EGFR, PI3K, and AKT as well as inactivating mutations of tumor suppressor PTEN or TSC1/2 causes the activation of the mTOR signaling pathway in a wide range of cancers [11,12]. However, the mTOR inhibitor sirolimus (rapamycin) is cytostatic but not cytotoxic. Although rapamycin achieved limited success in a few tumor syndromes such as TSC [36], most of the tumors are refractory to rapamycin treatment. Better or alternative regimens are therefore sought after. mTOR-mediated translation initiation is augmented in many cancers. The antineoplastic activity of eFT226, an inhibitor of the translation initiation factor eIF4A1, has been reported in a variety of cancer cells [25], eFT226 recently became the first rocaglate to enter clinical evaluation for advanced solid tumors in humans (ClinicalTrials.gov: NCT04092673) [23]. In the present study, *Tsc2*- and *Pten*-deficient cells were more sensitive than WT cells to eFT226 treatment. eFT226 significantly blocked tumor growth with limited drug toxicity in nude mice bearing *Tsc2*-deficient MEFs and the HCC cells PLC/PRF/5.

eFT226 inhibits translation initiation through forming a ternary complex with eIF4A and AGAGAG polypurine RNA oligonucleotides, preventing eIF4A1 releasing from the polypurine RNA motif [23,37,38]. In our study, eFT226 inhibits the eIF4F complex of *Tsc2*- and *Pten*-deficient MEFs without affecting the expression of eIF4A.

Cell death and the cell cycle function in a coordinated manner. Cell cycle arrest induces RIP3 phosphorylation and enhances necroptosis [39] The cyclinB1/CDK1 complex is the essential player in G2/M transition. eFT226 induced G2/M arrest, presumably by downregulating Cdc25c, CDK1, and cyclinB1 in our study. The G2/M phase arrest caused by eFT226 in *Tsc2*- and *Pten*-deficient MEFs could be partially attenuated by Nec-1. eFT226 exerted broad anti-tumor activity across different cancer cell lines via the induction of apoptosis [23,40]. In this study, eFT226 induced necroptosis by upregulating the expressions of RIPK1, RIPK3, and MLKL in *Tsc2*- and *Pten*-deficient MEFs. Nec-1 blunted eFT226-induced necroptosis and the elevation of RIPK3 and p-MLK. Even though we do not know the reason contributed to the discrepancy between previous reports and our study, eFT226-mediated apoptosis or necroptosis may be cell type-dependent. Nevertheless, eFT226 may be a novel regimen for the treatment of common cancers such as endometrial cancer and glioblastoma and rare diseases such as TSC and Cowden Syndrome.

mTOR inhibition by rapamycin and rapalogs mainly accumulates cells in the G1 phase of the cell cycle [41], while eFT226 induces G2/M phase arrest [23]. CDKs are master regulators of cell division and their inhibitors block cells at different stages of the cell cycle [42]. It is of interest to test whether combinations of mTOR inhibitors, CDK inhibitors, and translation initiation factor inhibitors trigger a durable cell cycle arrest for the treatment of cancer cells.

In conclusion, eFT226 preferentially inhibited *Tsc2*- and *Pten*-deficient cells by inducing necroptosis and G2/M arrest. In addition, eFT226 blocked *Tsc2*-deficient tumor growth. Inhibiting the activity of the eIF4F complex may represent a cancer vulnerability that could be clinically exploited to overcome chemoresistance and tumor heterogeneity. The present study provides preclinical evidence for the potential clinical application of eFT226 in hyperactive mTOR-associated tumors.

## Data Availability

The data is contained within the article.
